# A series of small-scale atmospheric datasets observed in south of Java, Pangandaraan Bay, Indonesia

**DOI:** 10.1016/j.dib.2023.109609

**Published:** 2023-09-22

**Authors:** Noir P. Purba, Ibnu Faizal, Hind Azidane, Alexander M.A. Khan, Lantun P. Dewanti, Sanny T. Utami, Kalysta Fellatami

**Affiliations:** a Marine Conservation Programme, Faculty of Fishery and Marine Science, Padjadjaran University, Jl. Raya Bandung-Sumedang Km. 21, Bandung, West Java, Indonesia; b Department of Marine Science, Faculty of Fishery and Marine Science, Padjadjaran University, Jl. Raya Bandung-Sumedang Km. 21, Bandung, West Java, Indonesia; c Department of Geology, Faculty of Sciences, Ibn Tofail University, B.P. 133 Kenitra, Morocco; d Department of Fishery, Faculty of Fishery and Marine Science, Padjadjaran University, Jl. Raya Bandung-Sumedang Km. 21, Bandung, West Java, Indonesia; e College of Fisheries, Ocean University of China, Qingdao, China

**Keywords:** Regional climate, Wind pattern, Asian-Australian monsoon, Weather station

## Abstract

This paper presents a collection of small-scale atmospheric datasets obtained from a PCE-FWS 20 N weather station in Pangandaraan, a region situated in the southern part of Java Island. The datasets cover a period from March 2022 to April 2023, with hourly measurements of air temperature, humidity, wind speed, wind direction, and daily rainfall. The instrument was cleaned and calibrated every three months according to the manufacturer's guidelines. Every week the data was downloaded from the memory card, resulting in a total of 48,468 data points available in a publicly accessible repository. The collected data were organized into .csv format and visualized to facilitate analysis. Our study aims to explore the microclimate of Pangandaraan over an extended period and highlights its potential applications in various fields, such as applied oceanography, meteorology, fishing grounds, and agriculture.

Specifications Table

**Table:** 

Subject	Earth and Planetary Science
Specific subject area	Weather data, seasonal wind, micro-climate
Type of data	Table, Figure, Chart, Graph
How the data were acquired	Weather station PCE-FWS 20 N. Detail information can be seen at https://www.pce-instruments.com/english/slot/2/download/5933564/man-weather-station-pce-fws-20n-en_1397028.pdf
Data format	Raw data
Description of data collection	Weather station PCE-FWS 20 N collected data from March 2022 to April 2023. Six parameters are presented here: air temperature, humidity, relative pressure, wind speed, wind direction, and daily rainfall. The data format is a .csv file converted into Excel sheets.
Data source location	This data was collected with detailed information below: • Institution: The University of Padjadjaran (UoP) and KOMITMEN Research Group • City/Town/Region: PSDKU Pangandaraan, The University of Padjadjaran, Jl. Cintaratu, Cintaratu, Kec. Parigi, Kab. Pangandaraan, Jawa Barat 46393 • Country: Indonesia • Latitude and longitude: 7.649162°S and 108.508981°E • 48468 data from March 2022 to April 2023 were collected from Weather station PCE-FWS 20N
Data accessibility	The data is hosted on Mendeley (www.mendeley.com). Repository name: Mendeley Data (https://data.mendeley.com) Data identification number (doi): 10.17632/w3ptrd25yt.4 Direct URL to data: https://data.mendeley.com/datasets/w3ptrd25yt/4.

## 1 Value of Data

• The weather data presented in this work enhance the knowledge of atmosphere-land-ocean interaction for climate change adaptation. By studying the small-scale atmospheric datasets from Pangandaraan, valuable insights can be gained into the complex dynamics of the local climate system.
• Precise and long-term data is used to monitor the coastal weather and benefit stakeholders in managing the regions, including Marine Protected Areas (MPA), tourist activities, and other coastal activities.
• Finally, with precise, efficient, low-cost instruments, other regions can adopt this method to monitor the environment. By employing this approach, other regions can gather valuable environmental information to support local mitigation and management strategies.

## 2 Objective

In the era of climate change, weather stations play a crucial role in monitoring and studying local climate to enhance understanding of long-term climate trends [1]. By collecting and analyzing the data obtained from weather stations, scientists can make accurate predictions about the impacts of climate change and develop strategies to mitigate its effects. Weather stations provide valuable insights into the complex relationship between global warming and local weather conditions, enabling us to comprehend better and address the challenges posed by climate change.

The objective of presenting data from a weather station is to collect accurate and timely information about various atmospheric conditions and elements, especially in the Pangandaraan region. This datasets assist the government, scientists, and society to predict atmospheric conditions [2]. In a long-term basis, this information is crucial for understanding daily weather conditions and climate change trends. Moreover, it enhances our understanding of weather phenomena and their impacts, including predicting flooding, wind energy potential, evaporation for agriculture, and fishing activities. The dataset is made available online for free and can be used for further analysis.

## 3 Data Description

Pangandaraan is a region located in the southern part of West Java Province. To the south, this area directly faces the Indian Ocean where phenomenom such as monsoon, upwelling, and ocean eddies are prevalent [3,4]. The presence of the Indian Ocean plays a crucial role in shaping the local climate and weather patterns. Periodic monsoon winds affect the region throughout the year [5]. Typically, winds with higher humidity levels blow from Asia toward Australia from December through February. Then, drier winds from Australia's southeasterly direction blow from June through August.

The dynamic interaction between land-ocean-atmosphere has a significant impact on the weather and climate conditions in this regions due to its proximity to the the Indian Ocean [6]. A new Marine Protected Area (MPA) has been designated for this region, which is also renowed as a fishing ground. Fishermen rely on weather and wave indicators to locate fish. Therefore, the installation of a weather station is considered highly benefical for enhancing climate prediction and weather conditions avalaible to tourists, local government, and fishermen.

To support the objectives of this study, a weather station was built in the region that has complex atmospheric conditions. Previous findings have demostrated the use of weather stations in various areas such as agricultural, industrial, renewable energy, and weather prediction applications [7,8]. In this paper, a year of data from PCE-FWS 20N was presented. The weather station was specifically configured to collect crucial meteorological parameters, including air temperature, relative air humidity, relative pressure, wind speed, wind direction, and daily rainfall every hour. The data series are statistically analyzed and visually presented. Finally, the open-access repository provides the original with providing hourly data measurements.

The open repository consists two folders, which include figures and raw datasets [9]. The dataset file, titled “AWS Dataset Pangandaraan.xlsx”, contains 10 columns. The first three columns are time attributes while the other six contain atmosphere datasets. Wind direction terminology follow the industry manufacture guidance. Each parameters consist of 8085 data points with minimum, maximum, and average values included at the bottom of the column (Table 2). Users can choose one or more parameters for calculation or analysis as needed. The second folder, named “Picture” contains three figures including the monthly distribution of datasets, temporal data, and wind rose (Fig. 2, Fig. 3, Fig. 4). Additionally, users can filter data using the feature in excel sheet to extract specific time ranges for analyzing various phenomena correlated with atmosphere data around Pangandaraan, Indonesia.

The collected data series were subjected to statistical analysis to derive meaningful insights. Additionally, visual representations, such as graphs, charts, or maps, were employed to enhance the understanding and interpretation of the data [10]. These visual presentations effectively convey the patterns and trends observed in the weather data.

## 4 Experimental Design, Materials and Methods

### 4.1 Experiment setup

**Fig. 1:**
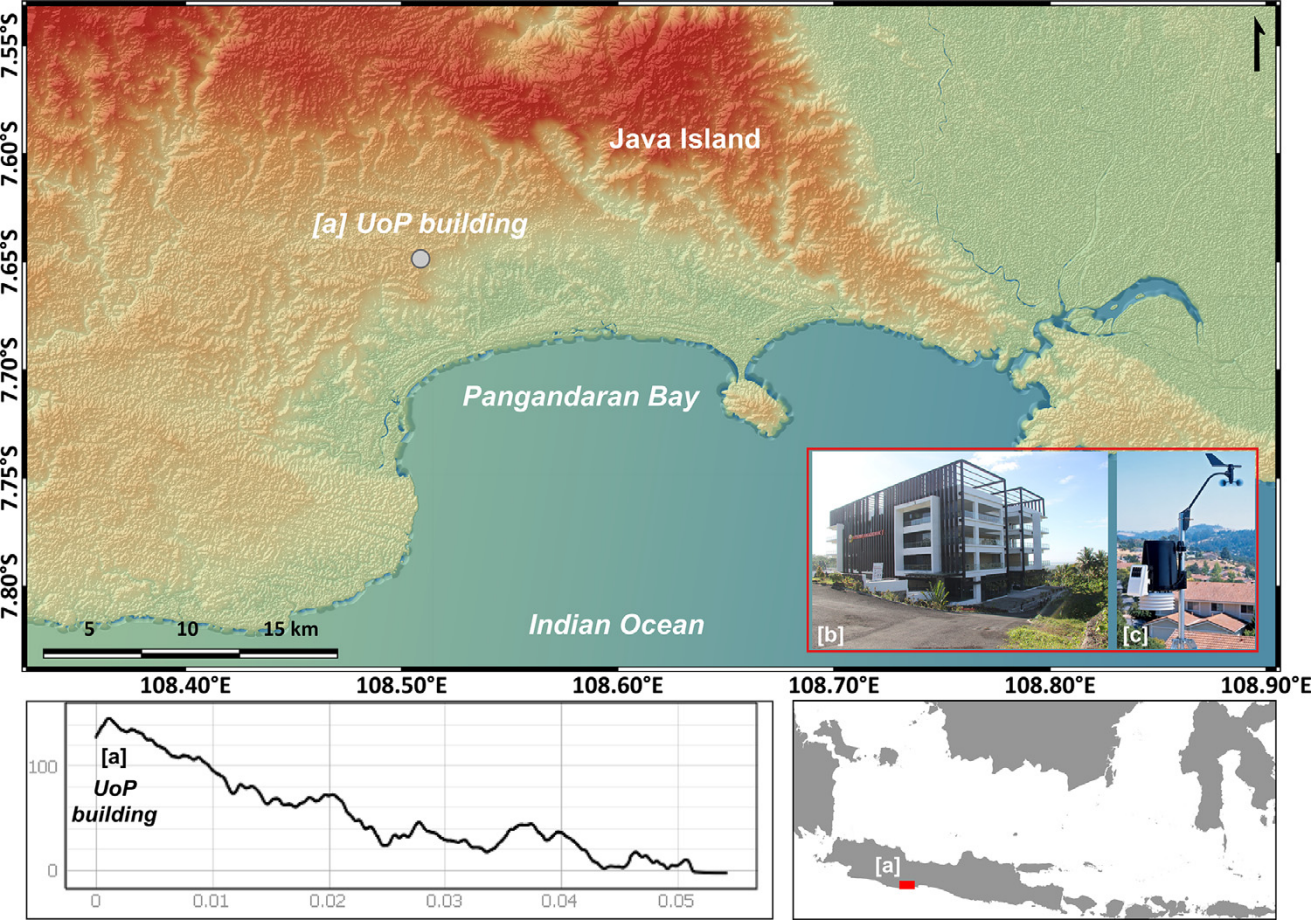
(top) Weather station located in the south of West Java region a) UoP-PSDKU building at Pangandaraan district, b) rooftop view of PSDKU building, and c) Weather station PCE-FWS 20N located on the top of the building. (bottom) The elevation from the PSDKU UoP building to the coastal area.

The weather station used in this study was installed at the PSDKU Building in the Cintaratu District. It was positioned at the highest point of the building, approximately 25 m above ground level. Specifically, the instrument is located at an elevation of 141 m above Mean Sea Level (MSL) at coordinates 7.649162°S–108.508981°E (Fig. 1).

The weather instrument is Automatic Weather Station (AWS) PCE-FWS 20N type. Full Specification of the device is provided in the manual document (https://www.pce-instruments.com/english/slot/2/download/5933564/man-weather-station-pce-fws-20n-en_1397028.pdf). PCE-FWS 20 N is a professionally calibrated weather station which includes sensors for measuring the following parameters:

1. Air temperature: This sensor measures the air temperature in the surrounding environment.
2. Humidity: The humidity sensor measures the relative humidity, indicating the air's moisture content.
3. Relative pressure: This sensor measures the atmospheric pressure relative to a reference pressure. The absolute air pressure is combined with the altitude information to calculate relative air pressure. This calculation helps to account for the influence of altitude on the atmospheric pressure measurements.
4. Wind speed: The wind speed sensor is responsible for measuring the speed or velocity of the wind.
5. Wind direction: This sensor determines the direction from which the wind is blowing.
6. Daily rainfall: The rainfall sensor measures the amount of precipitation over 24 h.

Table 1 provides further details and specifications of these parameters, including measurement range, accuracy, and resolution.

**Table 1:** Weather Station PCE-FWS 20N parameters.

No.	Air Temp.(°C)	Humidity (%)	Relatif Pressure (HPa)	Wind Speed (km/h)	Wind Direction	Daily Rainfall (mm)
Range	−40 to 60 °C	1 to 99 % HR	N/A	0…50	All dir.	0…9999 mm
Resolution	0.1 °C	NA	N/A	NA	NA	0.3 mm
Accuracy	± 1 °C	± 4 % RH	N/A	± 1 m/s	NA	± 6 %

The station is designed as wireless equipment with a cable connected to a transmitter powered by a solar panel and batteries. The weather station operates at a transmission frequency of 868 MHz. Both the visualization console and the station are located on the third floor and are monitored daily. Data is downloaded every week and to ensure the accuracy and performance of the weather station, regular manual cleaning is conducted every three months following industry manufacturer standards.

### 4.2 Method

Data was collected at hourly intervals from March 28, 2022, to April 4, 2023, covering four distinct monsoon seasons, including the northwest monsoon (December to February), transitional monsoon 1 (March to May), southeast monsoon (June to August), transitional monsoon 2 (September to November). The historical dataset contains some missing data for the measured period. Out of the total 12 parameters recorded, this paper presents data for six key parameters: Air Temperature, Humidity, Relative Pressure, Wind Speed, Wind Direction, and Daily Rainfall. The amount of data for each parameter per month is provided below (Table 2; dataset repository). Notalbly, there is missing data from the AWS measurements including a period from February 7, 2023, at 15:18 until February 16, 2023, at 14:50. Then, starting from March 6, 2023, at 15:23 until March 28, 2023, at 6:36. Additionally, there is a gap from March 28, 2023, at 15:36 until March 31, 2023, at 23:36. We did not apply any specific treatment to address missing data. Instead, we provided the raw data as collected, without imputing or filling in any gaps. The dataset repository includes detailed data for each parameter, enabling researchers to access and analyze the specific data points collected during the study period.

**Table 2:** Data statistics for each parameter.

No.	Air Temp. ( °C)	Humidity (%)	Relative Pressure (PA)	Wind Speed (km/h)	Wind Direction	Daily Rainfall (mm)
Min.	20.800	52.000	1005.7	0	–	0
Max.	33.000	99.000	1017.6	23.400	–	38.1
Ave.	25.542	84.081	1011.8	4.401	–	0.59

**Fig. 2:**
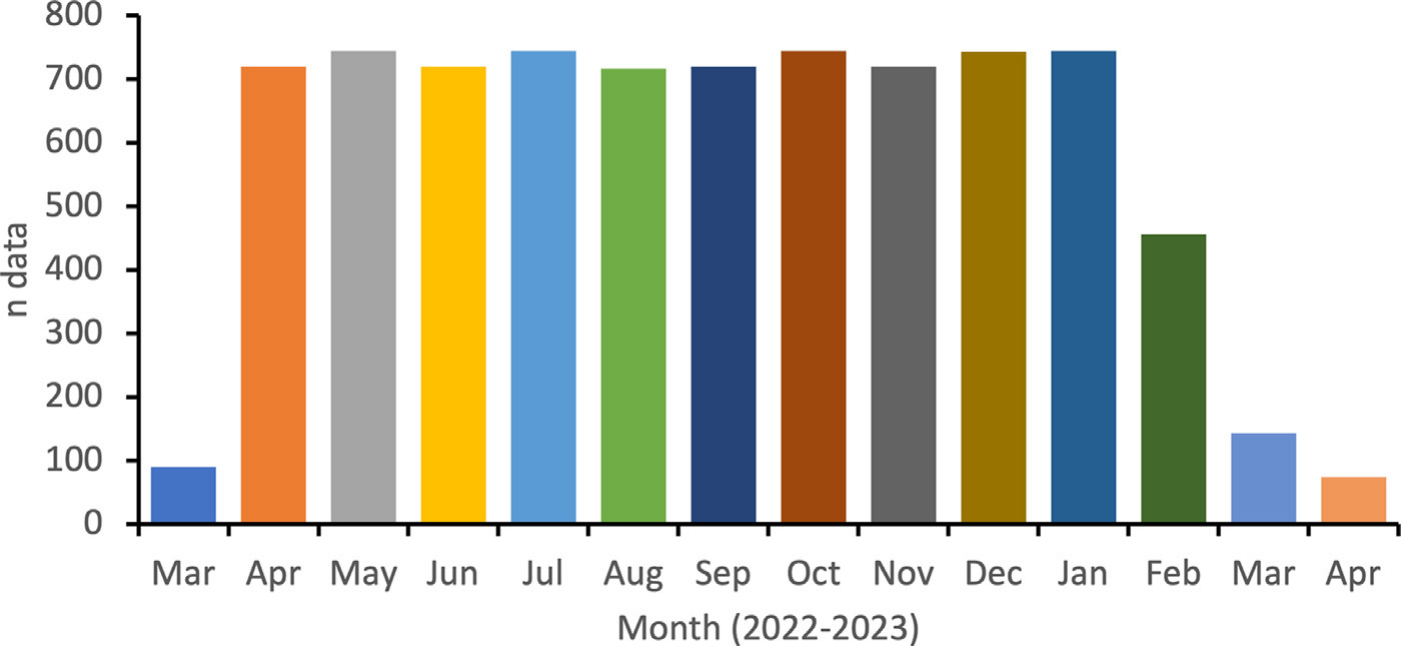
Monthly data for each parameter. Each parameter has the same amount of data.

**Fig. 3:**
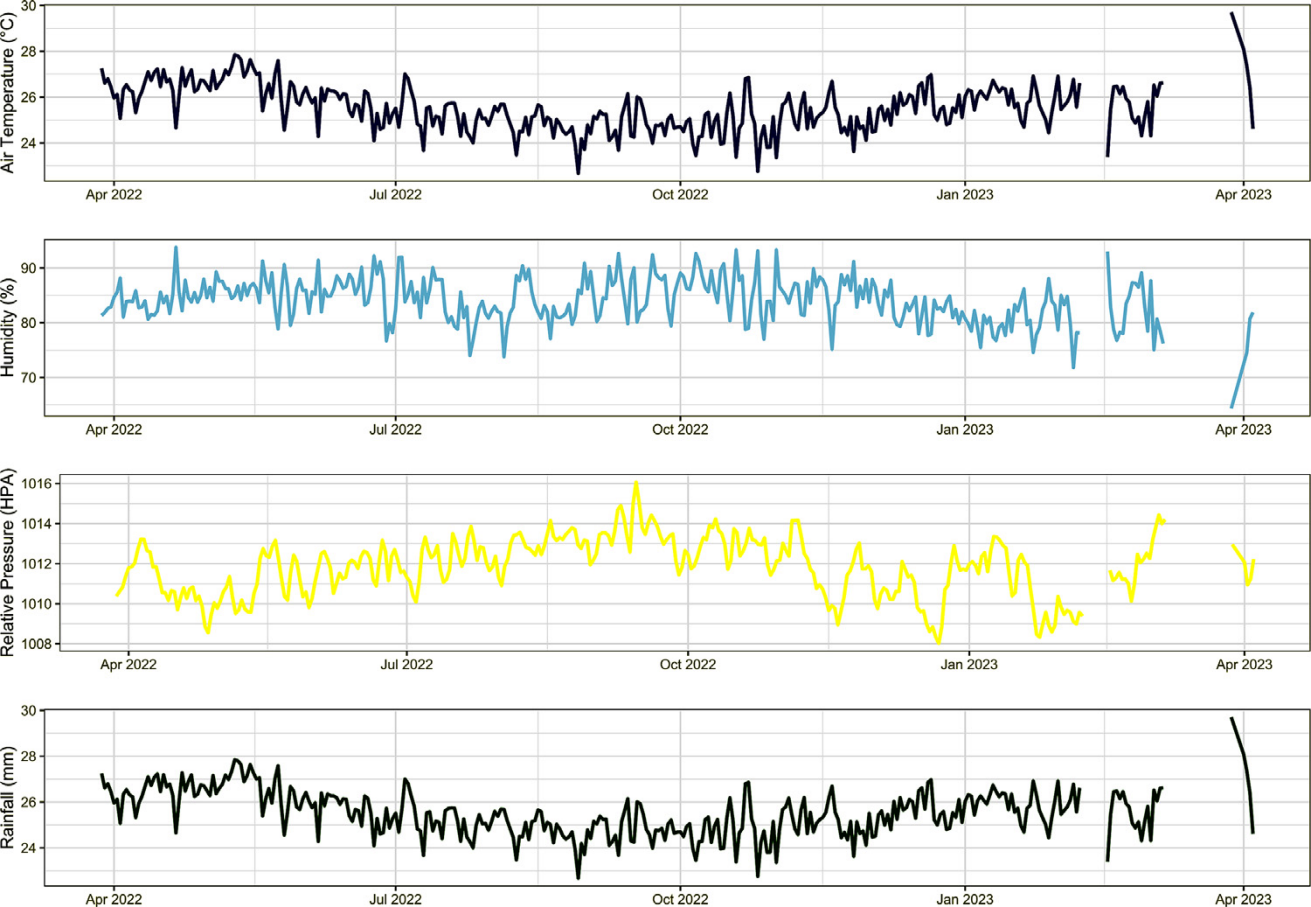
Daily average data from four parameters. Blank line is missing data.

**Fig. 4:**
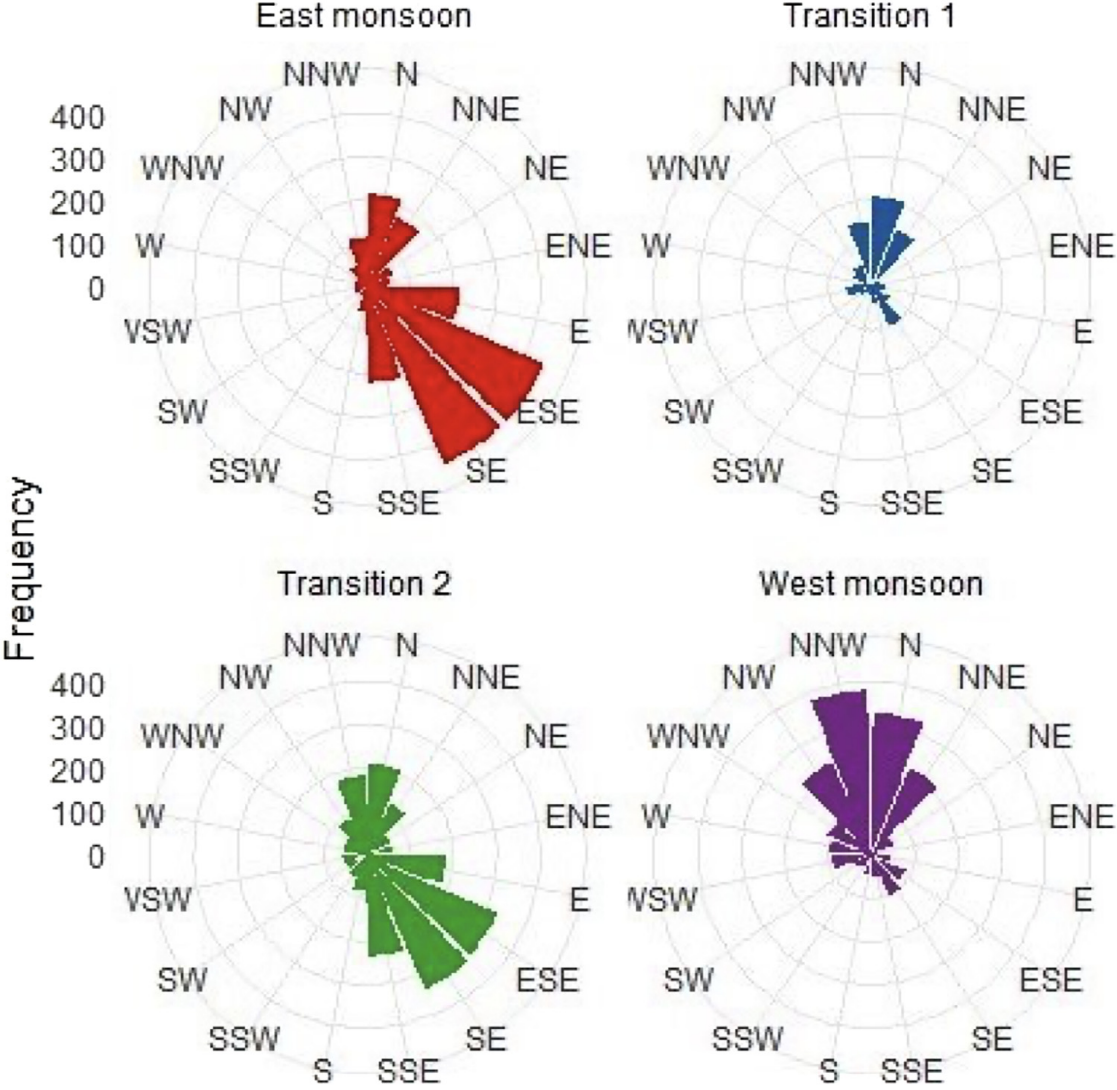
The wind rose graph and wind velocity.

The data from AWS is then sorted and used to generate standard statistics (Table 2). The raw data presented in the repository is available in .csv format. Initially, we have created daily average figures (Fig. 3) and wind direction is seasonality (Fig. 4). The header typically includes date and time, interval data, and parameter values. The time setting is in local time (GMT+7). The data is stored in open-access repository data (Mendeley Data: https://data.mendeley.com/datasets/w3ptrd25yt/4).

The data for each parameter, such as air temperature, humidity, relative pressure, wind, and daily rainfall, were initially averaged on a daily basis (Fig. 3). The daily averages provide a comprehensive overview of the parameter values throughout the study period, highlighting any potential trends or patterns.

Fig. 4 combines the wind rose graphs and wind velocity information in a single figure. The wind rose graphs depict the distribution of wind directions, displaying the predominant wind patterns observed in the study area. The wind velocity information complements the wind rose charts, indicating the intensity or speed of the wind corresponding to each direction.

The temperature data analysis reveals that the highest recorded temperature during the study period was 33.0 °C, which occurred in May 2022. Conversely, the lowest temperature was recorded in October 2022, reaching 20.80 °C. The average temperature for the period was approximately 25.542 °C. Regarding humidity, the range fluctuated between 52 and 99 %, with the highest levels observed in July and May 2022 and the lowest and highest levels occurring within the same month. The average humidity level was 84.081 %.

The relative pressure ranged from approximately 1005,7 to 1017 Hpa, with an average value of around 1011.8 Hpa. The highest pressure occurred in September 2022, while the lowest was observed in December 2022. The average daily rainfall approximately 0.59 mm, with values ranging from 0 to 38.1 mm. Occasional zero rainfall ocured throughout the year, with the highest recorded rainfall in September 2022. Wind direction primarily came from the Southeast (S.E.) and Northwest (N.W.), with an average speed of around 4.40 km/h. While zero wind speed rarely occurred, the highest recorded wind speed was approximately 23,4 km/h on June 2022.

## Ethics Statements

The authors declare that the present work did not include experiments on human subjects and/or animals.

## CRediT authorship contribution statement

**Noir P. Purba:** Writing – original draft, Supervision, Validation. **Ibnu Faizal:** Methodology, Writing – review & editing. **Hind Azidane:** Validation, Conceptualization, Formal analysis. **Alexander M.A. Khan:** Investigation, Writing – review & editing. **Lantun P. Dewanti:** Data curation, Project administration. **Sanny T. Utami:** Visualization, Software. **Kalysta Fellatami:** Funding acquisition, Data curation, Resources.

## Data Availability

UoP Pangandaran Weather Station Dataset (Original data) (Mendeley Data)
